# Favorable Effects of Virgin Coconut Oil on Neuronal Damage and Mortality after a Stroke Incidence in the Stroke-Prone Spontaneously Hypertensive Rat

**DOI:** 10.3390/life12111857

**Published:** 2022-11-11

**Authors:** Rodel Jonathan Santos Vitor, Ryota Tochinai, Shin-Ichi Sekizawa, Masayoshi Kuwahara

**Affiliations:** 1 Laboratory of Veterinary Pathophysiology and Animal Health, Department of Veterinary Medical Sciences, Graduate School of Agricultural and Life Sciences, The University of Tokyo, Tokyo 113-8657, Japan; 2 Department of Biology, College of Science, De La Salle University, Manila 1004, Philippines

**Keywords:** neuronal damage, SHRSP model, stroke, survival rate, virgin coconut oil

## Abstract

Stroke is consistently one of the top ten causes of morbidity and mortality globally, whose outcomes are quite variable, necessitating case-specific management. Prophylactic diets before the onset of stroke have been implicated to work. In this research, the effects of virgin coconut oil (VCO) on stroke were evaluated using a stroke-prone spontaneously hypertensive rat (SHRSP) model. Eight-week-old SHRSPs were subjected to the repeated oral administration (5 mL/kg/day) of either 1% Tween 80 (group A) or VCO (group B). An early stroke onset was observed due to hypertension that was aggravation by the administration of 1% NaCl in water ad libitum. The following data were collected: the days until stroke occurred, the survival rate until the animal died, and blood pressure (BP) every two weeks using the tail-cuff method. After necropsy, the organs were harvested, and the brain was processed for a routine histopathological analysis. VCO delayed the incidence of it and prolonged their survival. Compared to group A, group B showed a significantly lowered BP by 20 mmHg at four weeks after the start of VCO treatment. Lastly, the brain histopathology showed that the structurally damaged areas were smaller in group B than they were in group A. The VCO could have protective effects on the brain before and even after stroke incidence.

## 1. Introduction

Stroke is defined as an episode of acute neurological dysfunction that is presumed to be caused by ischemia or hemorrhage, persisting for ≥24 h or until death [1,2], and it is seen with rapidly developing clinical signs that are either focal or global [3]. It has been classified as the third and second leading cause of disability and mortality, respectively, worldwide according to the 2017 update of the American Heart Association of heart diseases and stroke [4,5]. It was previously thought that it was predominant in low- and middle-income countries [6], but it was observed in 2013, that the highest prevalence of ischemic stroke was seen in developed countries (1015–1184 cases per 100,000 people) versus developing countries (339 per 100,000 population) [4]. Furthermore, subsequent deaths have been expected to climb from 6.3 to 7.8 million from 2015 to 2030 [7]. Though different risk factors have been attributed to the pathogenesis of stroke, the most prevalent ones includes hypertension, diabetes mellitus, and smoking, which lead to its onset at least 50% of the time [3].

Stroke management methods currently include invasive surgical procedures (i.e., craniotomy, hemicraniotomy, and thrombectomy) [8] and the use of anticoagulants (number of needed treatments [NNT] = 25) [9,10], antiplatelets (NNT = 79) [11], and fibrinolytics (NNT = from 3.6 to 19.3, with lower values for patients who were treated earlier in the course of disease) [12]. Natural products, which are potential therapeutic alternatives for stroke patients are still the adjunctive therapy options as they have proven antithrombotic, antioxidant, hypoglycemic, hypocholesterolemic, and hypotensive properties. These properties may improve the patients’ circulation, toxin dissolution, and connective tissue repair, which are common sequelae of ischemia- or hemorrhage-induced stroke [13].

Virgin coconut oil (VCO), a saturated medium-chain fatty acid (MCFAs), is growing in popularity both as a functional food and as a possible treatment for several diseases [14]. VCO, unlike long-chain fatty acids (LCFAs), is readily absorbed in the gut [15]**,** and it is biotransformed in the liver to become acetoacetate that can be further metabolized into BHB [16]. In recent studies, it has been shown that β-hydroxybutyrate (BHB), which is a by-product of lipid metabolism, has protective effects against stroke and degenerative diseases by acting on the hydroxyl-carboxylic acid receptor 2 (HCA_2_) [17]. These HCA_2_, when they are activated, induce a neuroprotective phenotype of Ly-6CLo, monocytes and/or macrophages which infiltrate the ischemic brain which depends on prostaglandin D_2_ (PGD_2_) production by cyclooxygenase-1 (COX1) and the hematopoietic PGD_2_ synthase [16]. Furthermore, it has been assessed that ketones may also be used as an alternative source of energy during times of deficit of carbohydrate sources [18]. VCO has been shown to have anti-oxidative [19], anti-inflammatory [20,21], anti-pyretic [22], anti-nociceptive [21], anti-microbial [23], anti-hyperglycemic [24], anti-hypercholesterolemic [25], anti-atherogenic [26], hematologic [24], hepatoprotective [27,28]**,** and cardioprotective [29] properties. Moreover, its role as an adjunctive treatment for Alzheimer’s disease [30] and HIV [31] has also been evaluated.

The outcome of stroke is highly variable, and there are only a limited number of medications that have been accepted to reduce its deleterious effects. Since the consequences of stroke are mainly attributed to the excitotoxic effect of glutamate release, leading to an area of infarction, it is warranted to assess the possible neuroprotective effects of VCO after such an episode. This is based on the underlying mechanism that VCO may be used as an alternative energy source and due to its anti-oxidative properties. At present, the burden of stroke is still high, with the research stating that it may become the number one cause of disability and mortality in the few years to come. However, the current treatments that are available are about preventing the deleterious effects after a stroke incidence with the NNT ranging from three to eighty. As such, an evaluation of the possible neuroprotective treatments is needed to prevent the extensive damage that is caused by it. Considering the published effects of VCO supplementation in other diseases and conditions, as mentioned above, we evaluated its potential role in stroke evolution when it was induced by a high-salt treatment to stroke-prone spontaneously hypertensive rats (SHRSPs), and we expected to observe neuroprotection and the permanent prevention of disability or death. 

## 2. Materials and Methods

### 2.1. Ethics

All of the experiments using rats were conducted in accordance with the Animal Experimentation Guidelines of the University of Tokyo and were approved by the Institutional Animal Care and Use Committee of the Graduate School of Agricultural and Life Sciences at the University of Tokyo (P19-063H02; approved on 27 August 2019).

### 2.2. Animals

Eight-week-old male stroke-prone spontaneously hypertensive rats (SHRSP/Izm) (Japan SLC, Inc., Shizuoka, Japan) were randomly assigned into two groups: group A (*n* = 10) which was orally administered with 1% tween 80, and group B (*n* = 10) which was orally administered with VCO both at 5 mL/kg starting from 9 weeks of age. Tween 80 was used as control as it mimics the viscosity of VCO. They were placed individually in standard-sized cages, fed with rat pellet chows (the crude protein was not less than 23.0%, the crude fat was not less than 6.5%, and the crude fiber was not more than 4.5%) and distilled water ad libitum until the start of experimentation. They were kept at between 20 and 22 °C with a humidity of 74 ± 2 and on a 12 h light:12 h dark cycle. All of the cages, feeders, and water bottles were cleaned at least once a week and disinfected. All of the animals were acclimatized for one week prior to the experimentation. At the start of experimentation, the distilled water was replaced with 1% NaCl water which was provided as libitum.

### 2.3. Treatment

The VCO was purchased from a local supermarket in the Philippines. The analysis of the bioactive components of the VCO which was prepared using the cold method has been reported by Srivastava et al. [32]. The Tween 80 was obtained from Sigma-Aldrich.

### 2.4. Incidence and Survival Rates

The incidence of stroke was computed from the day salt-water was introduced until the animal was assessed to have experienced stroke using the neurological scoring developed by Bederson et al. [33] (Table 1). The rats were identified to have experienced stroke if the score based on the neurological scoring was 1 and above. Their survival, on the other hand, was computed from the time that they were classified as having experienced stroke until their death or until a humane endpoint had been reached. The humane endpoints were defined as when an animal had lost 10% of its body weight or if they showed ataxic movements, were motionless, or were moribund.

### 2.5. Systolic and Diastolic Blood Pressure and Heart Rate

Their blood pressures and heart rates were measured at the baseline (before the start of treatment) and every 2 weeks thereafter using the tail-cuff method (BP-98A, Softron Co., Ltd., Tokyo, Japan). Their blood pressures and heart rates were taken 3 times within 10 min, and the average value was recorded.

### 2.6. Average Food Intake, Body Weight, and Relative Organ Weights

Their average food intake and body weights were measured at the baseline and every 2 weeks thereafter. The animals were observed twice a day, and they were necropsied after death or sacrificed under isoflurane inhalation anesthesia if they reached the humane endpoints. The brain, heart, liver, right and left kidneys, and the spleen were collected and weighed upon necropsy, and the relative organ weights which were compared with the brain weights were obtained. These organs were selected as they may have been affected or could be affect the blood pressure as well as the pathogenesis of stroke, especially for the heart, kidneys, and spleen. On the other hand, the liver was collected as it may be used as a source of energy via the glycogenolytic pathway.

### 2.7. Histopathological Analysis of Brain Tissues

The brain was processed for routine histopathological analysis using H and E stain. In brief, the brain samples were cross-sectionally cut at the level of the bregma, and representative samples were examined for hypoxic neuronal cells. The number of hypoxic neuronal cells were counted in ten random fields (200×), and this was averaged per animal. Other notable features such as reactive gliocytes and the presence of cerebral and subarachnoid hemorrhages were likewise noted.

### 2.8. Statistics

The statistical significance was determined at *p* < 0.05 using the Kaplan–Meier survival curves which were assessed by a log-rank test for the incidence and survivability, while a *t*-test was used for the blood pressure, body weight, relative organ weights, and hypoxic neurons which are reported as the mean ± SD.

## 3. Results

### 3.1. Incidence and Survival Rates

**Figure 1:**
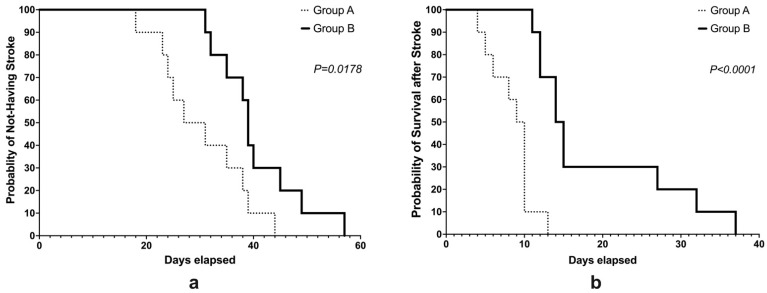
The incidence rate (**a**) and the survival rate (**b**) of stroke-prone spontaneously hypertensive rats (SHRSP) that were administered with either Tween 80 (group A) or VCO (group B).

All of the 20 rats that were used in the experiment were observed to have experienced stroke, with the first incidence having been observed at 18 days after the start of salt-loading. On the other hand, the last animal to be observed with stroke was at 57 days after the salt-loading stage. The Cox proportional hazards model revealed that the incidence of stroke in group A was significantly different when it was compared to that of group B which was treated with VCO (*p* = 0.0178) (Figure 1a). Similar results were observed in the survival of the animals after a stroke incident. The first death was observed 4 days after stroke, while the last animal died at 37 days after stroke. The Cox proportional hazards model showed that the survival in group A was significantly shorter when it was compared to that of group B (*p* < 0.0001) (Figure 1b).

**Figure 2:**
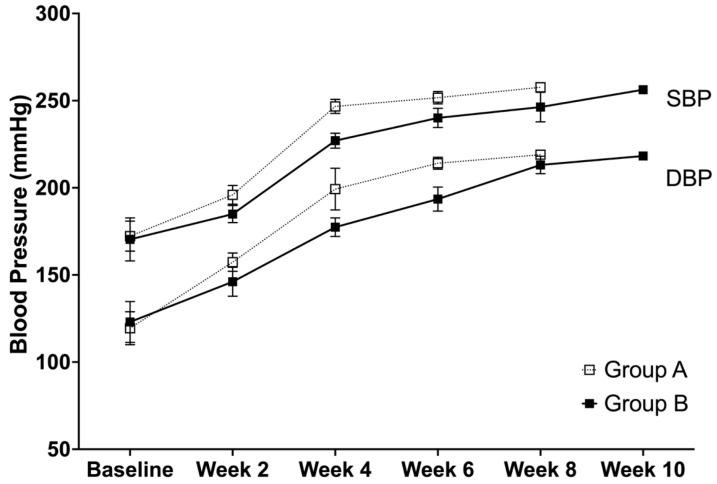
The systolic and diastolic blood pressure (mean ± SD) of SHRSPs that were administered with either Tween 80 (group A) or VCO (group B).

**Figure 3:**
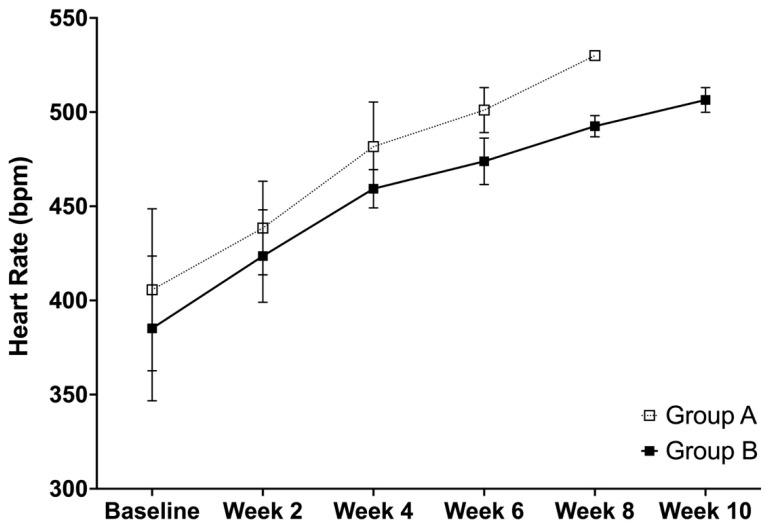
The heart rate (mean ± SD) of SHRSPs that were administered with either Tween 80 (group A) or VCO (group B).

In the succeeding discussions (Figure 2 and Figure 3), the animal withdrawals were observed due to the deaths of the animals, and the number of remaining animals were reduced at different time points. It was noted that all of the animals died after the tenth week of the experiment (Table 2). Thus, no comparison can be made between the groups at week 8 since as there was only one animal left at group A, and at week 10, all of the group A animals had already died. Further, no time effect analysis was made between weeks 6 and 8, and weeks 8 and 10 for group A.

### 3.2. Systolic and Diastolic Blood Pressure

The trend for both the systolic and diastolic blood pressure shows that there was a similar trend, in which an increase in the values were observed at every time point. However, the analysis showed that there were significant differences between group A and B for SBP at week 2 (*p* = 0.0001), week 4 (*p* < 0.0001), and week 6 (*p* = 0.0023), while no difference was observed at the baseline (*p* = 0.6995) (Figure 2). For group A, the time effects were significant between the baseline and week 2 and weeks 2 and 4 (both *p* < 0.0001), while no difference was observed between weeks 4 and 6 (*p* = 0.0519). On the other hand, for group B, the time effects were significant between the baseline and week 2 (*p* = 0.0008), weeks 2 and 4 (*p* < 0.0001), weeks 4 and 6 (*p* < 0.0001), and weeks 8 and 10 (*p* = 0.0447), while no difference was observed between weeks 6 and 8 (*p* = 0.0717).

The analysis showed significant differences between group A and B at DBP at week 2 (*p* = 0.0020), week 4 (*p* = 0.0001), and week 6 (*p* = 0.0001), while no difference was observed at the baseline (*p* = 0.4564) (Figure 2). For group A, the time effect was significant between the baseline and week 2 (*p* < 0.0001) and weeks 2 and 4 (*p* < 0.0001), but not between weeks 4 and 6 (*p* = 0.343). For group B, the time effect was significant between the baseline and week 2 (*p* = 0.0001), weeks 2 and 4 (*p* < 0.0001), weeks 4 and 6 (*p* < 0.0001), and weeks 6 and 8 (*p* < 0.0001), while no difference was observed between weeks 8 and 10 (*p* = 0.0770).

### 3.3. Heart Rate 

Similar to the trend in blood pressure, the heart rate shows an increase with the heart rates for both of the groups. Specifically, the analysis showed significant differences between groups A and B at weeks 4 (*p* = 0.0134) and 6 (*p* = 0.0028), while no differences were observed at the baseline (*p* = 0.2759) and week 2 (0.1952) (Figure 3). For group A, the time effects were found to be significant between weeks 2 and 4 (*p* = 0.0009), while no differences were observed between the baseline and week 2 (*p* = 0.0513) and weeks 4 and 6 (*p* = 0.1509). For group B, the time effects were significant between all of the comparisons at the baseline and week 2 (*p* = 0.0158), weeks 2 and 4 (*p* = 0.0005), weeks 4 and 6 (*p* = 0.0099), weeks 6 and 8 (*p* = 0.0007), and weeks 8 and 10 (*p* = 0.0024).

### 3.4. Average Feed Intake, Body Weight, and Relative Organ Weights

**Figure 4:**
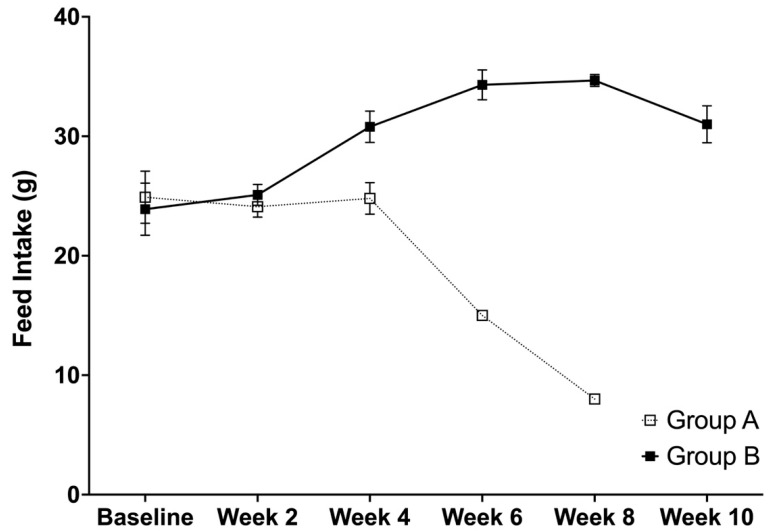
The average feed intake (g) (mean ± SD) of SHRSPs that were administered with either Tween 80 (group A) or VCO (group B).

It can be seen that there was a decrease in the feed intake after week 4 for group A and after week 8 for group B. These weeks corresponds with the identification of the rats as having experienced stroke. More specifically, the average feed intake between groups A and B were found to be different at weeks 2 (*p* = 0.0199), 4 (*p* < 0.0001), and 6 (*p* < 0.0001), while no difference was observed at the baseline (*p* = 0.3193) (Figure 4). For the effects of time, significant differences were observed in group A between weeks 4 to 6 (*p* = 0.0000), while non-significant differences were observed between the baseline and week 2 (*p* = 0.2964) and weeks 2 and 4 (*p* = 0.1785). For group B, the time effects were found to be significant between weeks 2 and 4 (*p* < 0.0001), weeks 4 and 6 (*p* < 0.0001), and weeks 8 and 10 (*p* < 0.0001), while no differences were observed between the baseline and week 2 (*p* = 0.1241) and weeks 6 and 8 (0.4236).

**Figure 5:**
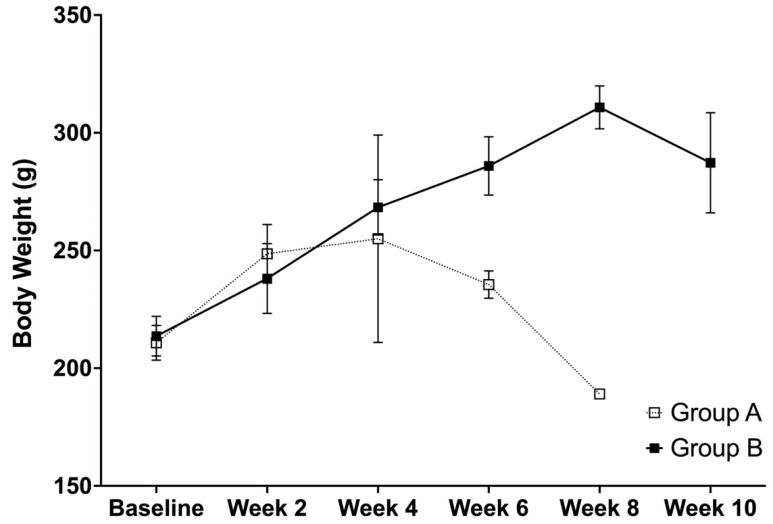
The body weight (g) (mean ± SD) of SHRSPs that were administered with either Tween 80 (group A) or VCO (group B).

The analysis showed a significant difference between the body weight of group A and B at week 6 (*p* < 0.0001), while no differences were observed at the baseline (*p* = 0.4397), week 2 (*p* = 0.1034), week 4 (*p* = 0.3687) (Figure 5). For group A, the time effects were significant between the baseline and week 2 (*p* < 0.0001), while non-significant differences were observed between weeks 2 and 4 (*p* = 0.6638) and weeks 4 and 6 (*p* = 0.4060). For group B, the time effects were significant between all of the comparisons at the baseline and week 2 (*p* = 0.0002), weeks 2 and 4 (*p* = 0.0001), weeks 4 and 6 (*p* = 0.0044), weeks 6 and 8 (*p* = 0.0001), and weeks 8 and 10 (*p* = 0.0147). There was a general trend of weight decline after week 4 for group A and week 8 for group B. This can be attributed to the decrease in the feed intake of animals which was observed to be associated with stroke, as can be observed from Figure 4.

**Figure 6:**
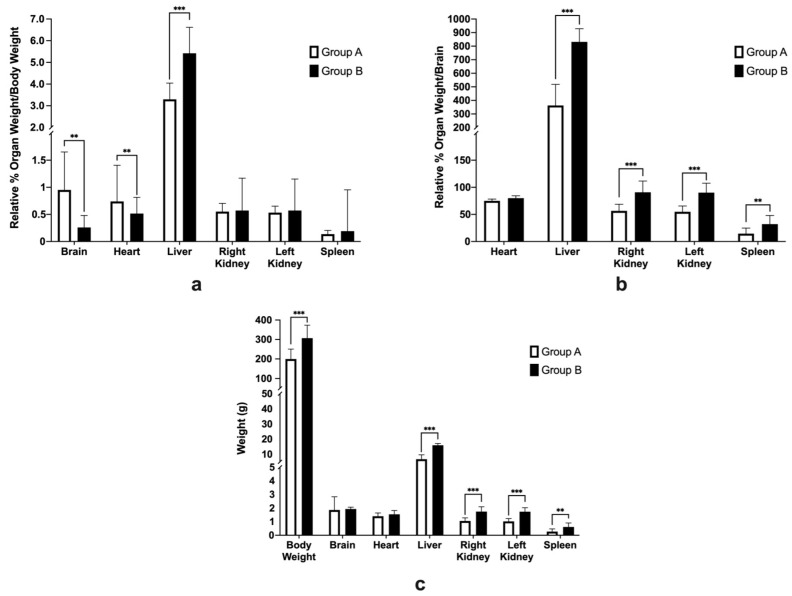
The relative % organ weights (mean ± SD) which were compared with (**a**) body weight and (**b**) brain, and (**c**) the body weight and organ weights at necropsy of SHRSPs that were administered with either Tween 80 (group A) or VCO (group B). ** *p* < 0.01, *** *p* < 0.001 (*t*-test).

Their relative organ weights were compared to the body weight, and also, the brain as there has been research that shows that brain does not decrease in weight drastically in comparison to the body weight [34]. Figure 6a shows the organ weights relative to the body weight; significant differences between group A and group B were observed for the brain (*p* = 0.0045), heart (*p* = 0.0067) and liver (*p* = 0.0002), but there were no differences for the right kidney (*p* = 0.7026), left kidney (*p* = 0.3573), or spleen (*p* = 0.1086).

Figure 6b shows the organ weights relative to the brain; significant differences between groups A and B were observed for the liver (*p* < 0.0001), right kidney (*p* = 0.0003), left kidney (*p* < 0.0001), and spleen (*p* = 0.0086), while a non-significant difference was observed for the heart (*p* = 0.3826)

It can be speculated that the observed significant differences of the relative organ weights for the brain and the liver when we were using the body weight as a reference could be attributed to the significant differences of the body weight at necropsy (*p* = 0.0007) since the brain weight at necropsy was not significantly different (*p* = 0.1964) (Figure 6c). The actual weight of the heart (*p* = 0.2418) was also not significant between groups A and B, while the liver (*p* < 0.0001), right kidney (*p* = 0.0001), left kidney (*p* < 0.0001), and spleen (*p* < 0.0067) were significantly different between groups A and B.

### 3.5. Histopathological Analysis of Brain Tissues

**Figure 7:**
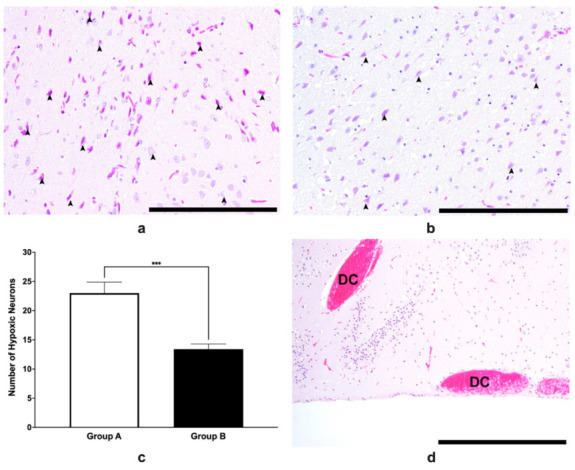
H and E staining in the SHRSP brain slices. The neocortex of SHRSPs that were administered with Tween 80 (group A) or VCO (group B) is shown in the upper left (**a**) or right (**b**), respectively. Group data show that the number of hypoxic neurons (mean ± SD) was significantly greater in group A than group B (**c**). In the parenchyma, dilated capillaries with RBCs were characteristic in SHRSPs without VCO treatment (**d**). Legend: arrowhead—hypoxic neurons; DC—dilated capillaries with RBCs. Bar scale—200 μm (**a**,**b**); bar scale—1000 μm (**d**). *** *p* < 0.001 (*t*-test).

As shown in Figure 7a,b, more hypoxic neurons were observed in group A than in group B, as confirmed by the group data (Figure 7c). There seemed to be more reactive astrocytes that were present in group A when it was compared with group B, which may signify that there was more damage in the former when it was compared with the latter since reactive astrocytes could signify structural reorganization as a consequence of brain hypoxia. Further hemorrhages, whether they are parenchymal or subarachnoid, were more predominant in the control group (Figure 7d). 

## 4. Discussion

In our current study, we have found favorable effects of VCO in an SHRSP model, specifically in delaying the incidence, increasing the survivability, possibly delaying the onset of hypertension in relation to stroke, and it also had some protective effects on the liver, kidney, and spleen. Virgin coconut oil is rich in lauric acid, a fatty acid with anti-oxidative properties [19]. It is rich in polyphenols which can increase the levels of antioxidant, thereby reducing lipid peroxidation and inflammation in VCO-treated mice [21]. The restoration of the antioxidant levels may be one of the mechanisms that can prevent further neuronal damage and avoid the subsequent depletion of monoamines, including dopamine [35]. Furthermore, recent studies have shown that VCO administration increased the hepatic GSH (reduced glutathione) levels, including the catalase and super oxide dismutase (SOD) activities [36,37]. Glutathione reductase, which reduces glutathione to GSH, has a protective mechanism by reducing the peroxide toxicity [38]. GSH is an important antioxidant, and it is a detoxifying molecule that helps to maintains the normal redox status in the body, with the catalase and SOD levels being an indication of better oxidative homeostasis in the tissues [39]. These results are supported by a study in which thiobarbituritic acid reactive substances (TBARs) and carbonyl adducts, which are indicators of lipid peroxidation, have been found to be decreased in VCO-fed rats [36]. 

It was evident that the animals were still observed to be hypertensive, however, the delay in the incidence of stroke as well as the prolonged survivability may suggest that the components of VCO may have effects on stroke prevention possibly due to the deferral of hypertension development. Further, the HR was seen to be continuously increasing with the increase in blood pressure in the current study, which is similar to the reports of Dickhout and Lee [40] which state that elevations in the HR are highly predictive of hypertension in spontaneously hypertensive rats (SHR). Though significant differences were observed for the blood pressure at different time points, the elevation of the blood pressure was still evident across the time points, which is different from the previous study by Nurul-Iman et al. [41] who reported that VCO has anti-hypertensive effects in rats who were fed with repeatedly heated palm oil. However, our results are similar in the recent placebo-controlled clinical study of Júnior et al. [42] which concluded that using VCO as an adjuvant in the management of hypertension in humans is not supported. One possible hypothesis showing the differences in the results of the experiments are the different models that were used in both of the animal studies, while the human studies included enrolled volunteers that were already hypertensive. In the present study, the administration of VCO prolonged the time for the animal to be observed as having experienced stroke, including increasing their survivability. The studies on the consumption of different fatty acids have been evaluated to be beneficial to the brain such as in neural development, neuroprotection, and in potential treatments for numerous neurological disorders [43]. Based on the current experiment, it can be said that VCO may have given neuroprotection properties. It was observed in an unpublished study of Vitor [44] that VCO was able to increase the high-density lipoproteins (HDL) while decreasing the total cholesterol, triglyceride, the low-density and very low-density lipoproteins, and the HDL ratio. In a study in utilizing vitamin E in preventing neuronal deaths in SHRSP/Izm rats, the authors found that the xanthine oxidase inhibitor or superoxidase dismutase (SOD) inhibited the deaths of the neurons either due to hypoxia or reperfusion injury [45]. VCO has been shown to have antistress and antioxidant effects. In one study, the authors were able to determine that VCO can raise the levels of SOD, which can reduce peroxidation and inflammation [21]. In the study, it is hypothesized that the survivability of the animals was linked with presence of antioxidant polyphenols in VCO which can stimulate the antioxidant levels to increase, thereby preventing inflammation and indirectly suppress the apoptotic pathway in the brain. Other studies have shown that notable improvements in mitochondrial function, a decrease in inflammation, and an increase in the expression of neurotrophins such as brain derived neurotrophic factor (BDNF) and basic fibroblast growth factor (bFGF) were observed [18,46].

The differences in the relative organ weights may be attributed to the function of each organ in neurodegenerative processes as well as in the animal model that was being used. Our data showed that the relative weights of the liver were significantly different between the treated and control groups. This can be attributed to the possibility that the liver in the control groups was being used to supply energy by the glycogenolytic pathway [47], while VCO may act as a supply of energy for those in the treatment group. Others have also suggested that VCO may be used as an alternative source of energy during depleted states by using the ketogenic pathway [17,48], which would explain the significant differences in the liver size. On the other hand, protective effects on the kidneys may also have been conferred by VCO since the salt-loaded water may have also produced an effect in the kidney. This is different from the study of Mary et al. [49] who reported that there were no deleterious effects on the kidneys after 3 weeks of 1% salt-loading in SHRSPs. However, since the experiment in the current study was performed for 10 weeks, with the first death occurring at 4 weeks after the start of the salt loading, the differences in the lengths of the experiments might be attributed to the possible differences in the kidney. Similarly, the spleen has recently been found to have an effect in hypertension pathogenesis by providing immune cells for blood pressure regulation [50]. Overall, VCO may help various organs to stay functional against hypertension, which is a main risk factor of stroke, however, further studies will be required to elucidate these mechanisms in detail.

The number of hypoxic neurons were lower in the group that were given VCO when they were compared with the group that were administered with Tween 80 (Figure 6b). This may indicate that there are neuroprotective effects that VCO may have conferred. Much of the neurological dysfunction that occurs in stroke, cerebral ischemia, and acute traumatic brain injuries is due to a secondary injury process involving glutamate-mediated excitotoxicity, intracellular calcium overload, mitochondrial dysfunction, and the generation of reactive oxygen species (ROS) [51] which lead to a sequelae of apoptosis. Neuronal apoptosis is one of the important contributing factors to neurological deficiencies, and this has been studied extensively [52]. In previous studies, the downregulation of key proteins such as TLR4/NFkappaB inhibits apoptosis and inflammation, thus decreasing the size of the ischemic penumbra [53]. In another study, neamine has been found to significantly decrease caspase-3 as well as decreased the lesion volume in the ischemic brain [54]. Caspase-3 has been identified as an essential pathway in apoptosis in animal models of ischemic stroke. Furthermore, studies have shown that both the intrinsic or extrinsic pathways are caspase dependent [55]. These studies showed that decreasing the levels of the different proteins induces a neuroprotective effect by attenuating apoptosis and decreasing the lesion volume and the histologic damage. In a recent study of Diestro et al. [56], they were able to show that the infarct size which was determined using the tetrazolium method as well as hemispheric edema were not significantly different between the rats that were treated with VCO versus the control ones, although the neurologic deficit scores 24 h after surgery were different. Further, reactive astrocytes have been observed in several studies to be main regulators in the brain inflammatory cascade, however, in pathological conditions such as hypoxic states, it can be neurotoxic as well [57]. Further studies which will elucidate the possible anti-apoptotic effects of VCO are warranted to verify this hypothesis.

Using SHRSPs is one of the most common models that are used in experimental animals to show the possible effects of the treatments for ischemic stroke. In the present study, it was demonstrated that the treatment of VCO to SHRSPs could have favorable effects on the stroke incidence and survivability as well as in the histopathological analysis of the brain. This shows that VCO could be neuroprotective in stroke cases. The limitations of the current study includes that it was conducted in a spontaneous model, and as such, molecular studies cannot be performed since the timing of stroke was not equal for each animal. As such, it is recommended that future studies using non-stroke-prone rats may also be used to possibly find possible other factors that may related to the protective mechanisms of stroke. Lastly, further studies in a surgical model are warranted to elucidate its protective mechanisms, specifically for the parameters that can be affected by the timing of stroke onset.

## Figures and Tables

**Table 1:** Neurological scoring used to determine whether the individuals had experienced stroke or not [33].

Score *	Description
0	No neurological deficit
1	Left forelimb flexion when suspended by the tail or failure to extend the right forepaw fully
2	Left shoulder adduction when suspended by the tail
3	Reduced resistance to lateral push toward the left side
4	Spontaneous movements in all directions with circling to the left exhibiting only if pulled by the tail
5	Circle or walk spontaneously only to the left
6	Walk only when stimulated
7	No response to stimulation
8	Stroke-related death

* Rats were identified to have experienced stroke if they were classified as having a score of 1 and above.

**Table 2:** The number of survived SHRSPs that were administered with either Tween 80 (group A) or VCO (group B).

Group	Baseline	Week 2	Week 4	Week 6	Week 8	Week 10
A	10	10	10	4	1	-
B	10	10	10	10	9	4
Total	20	20	20	14	10	4

## Data Availability

The raw data supporting the conclusions of this manuscript will be made available by the authors without undue reservation to any qualified researcher.

## References

[1] Sacco R.L., Kasner S.E., Broderick J.P., Caplan L.R., Connors J.J., Culebras A., Elkind M.S., George M.G., Hamdan A.D., Higashida R.T. (2013). An updated definition of stroke for the 21st century: A statement for healthcare professionals from the American Heart Association/American Stroke Association. Stroke.

[2] Coupland A.P., Thapar A., Qureshi M.I., Jenkins H., Davies A.H. (2017). The definition of stroke. J. R. Soc. Med..

[3] Truelsen T., Begg S., Mathers C.D., Satoh T. Global Burden of Cardiovascular Disease in the Year 2000; 2000. http://www.who.int/healthinfo/statistics/bod_cerebrovasculardiseasestroke.pdf.

[4] Benjamin E.J., Blaha M.J., Chiuve S.E., Cushman M., Das S.R., Deo R., de Ferranti S.D., Floyd J., Fornage M., Gillespie C. (2017). Heart disease and stroke statistics-2017 update: A report from the American heart association. Circulation.

[5] Feigin V.L., Norrving B., Mensah G.A. (2017). Global burden of stroke. Circ. Res..

[6] Thrift A.G., Cadilhac D.A., Thayabaranathan T., Howard G., Howard V.J., Rothwell P.M., Donnan G.A. (2014). Global stroke statistics. Int. J. Stroke.

[7] Go A.S., Mozaffarian D., Roger V.L., Benjamin E.J., Berry J.D., Blaha M.J., Dai S., Ford E.S., Fox C.S., Franco S. (2014). Heart disease and stroke statistics--2014 update: A report from the American Heart Association. Circulation.

[8] Hankey G.J., Sudlow C.L., Dunbabin D.W. (2000). Thienopyridine derivatives (ticlopidine, clopidogrel) versus aspirin for preventing stroke and other serious vascular events in high vascular risk patients. Cochrane Database Syst. Rev..

[9] Aguilar M.I., Hart R. (2005). Oral anticoagulants for preventing stroke in patients with non-valvular atrial fibrillation and no previous history of stroke or transient ischemic attacks. Cochrane Database Syst. Rev..

[10] Chao T.F., Liu C.J., Liao J.N., Wang K.L., Lin Y.J., Chang S.L., Lo L.W., Hu Y.F., Tuan T.C., Chung F.P. (2016). Use of oral anticoagulants for stroke prevention in patients with atrial fibrillation who have a history of intracranial hemorrhage. Circulation.

[11] Sandercock P., Gubitz G., Foley P., Counsell C. (2003). Antiplatelet therapy for acute ischaemic stroke. Cochrane Database Syst. Rev..

[12] Lansberg M.G., Schrooten M., Bluhmki E., Thijs V.N., Saver J.L. (2009). Treatment time-specific number needed to treat estimates for tissue plasminogen activator therapy in acute stroke based on shifts over the entire range of the modified Rankin Scale. Stroke.

[13] Maas M.B., Safdieh J.E. (2009). Ischemic stroke: Pathophysiology and principles of localization. Neurol. Board Rev. Man..

[14] Marina A.M., Che Man Y.B., Amin I. (2009). Virgin coconut oil: Emerging functional food oil. Trends Food Sci. Technol..

[15] Page K.A., Williamson A., Yu N., McNay E.C., Dzuira J., McCrimmon R.J., Sherwin R.S. (2009). Medium-chain fatty acids improve cognitive function in intensively treated type 1 diabetic patients and support in vitro synaptic transmission during acute hypoglycemia. Diabetes.

[16] Lim S., Chesser A.S., Grima J.C., Rappold P.M., Blum D., Przedborski S., Tieu K. (2011). D-beta-hydroxybutyrate is protective in mouse models of Huntington’s disease. PLoS ONE.

[17] Rahman M., Muhammad S., Khan M.A., Chen H., Ridder D.A., Muller-Fielitz H., Pokorna B., Vollbrandt T., Stolting I., Nadrowitz R. (2014). The beta-hydroxybutyrate receptor HCA2 activates a neuroprotective subset of macrophages. Nat. Commun..

[18] Maalouf M., Sullivan P.G., Davis L., Kim D.Y., Rho J.M. (2007). Ketones inhibit mitochondrial production of reactive oxygen species production following glutamate excitotoxicity by increasing NADH oxidation. Neuroscience.

[19] Famurewa A.C., Aja P.M., Maduagwuna E.K., Ekeleme-Egedigwe C.A., Ufebe O.G., Azubuike-Osu S.O. (2017). Antioxidant and anti-inflammatory effects of virgin coconut oil supplementation abrogate acute chemotherapy oxidative nephrotoxicity induced by anticancer drug methotrexate in rats. Biomed. Pharmacother..

[20] Keddy P.G., Dunlop K., Warford J., Samson M.L., Jones Q.R., Rupasinghe H.P., Robertson G.S. (2012). Neuroprotective and anti-inflammatory effects of the flavonoid-enriched fraction AF4 in a mouse model of hypoxic-ischemic brain injury. PLoS ONE.

[21] Zakaria Z.A., Somchit M.N., Mat Jais A.M., Teh L.K., Salleh M.Z., Long K. (2011). In vivo antinociceptive and anti-inflammatory activities of dried and fermented processed virgin coconut oil. Med. Princ. Pract..

[22] Intahphuak S., Khonsung P., Panthong A. (2010). Anti-inflammatory, analgesic, and antipyretic activities of virgin coconut oil. Pharm. Biol..

[23] Shilling M., Matt L., Rubin E., Visitacion M.P., Haller N.A., Grey S.F., Woolverton C.J. (2013). Antimicrobial effects of virgin coconut oil and its medium-chain fatty acids on Clostridium difficile. J. Med. Food.

[24] Handajani N.S., Dhamawan R. (2009). Effect of VCO to leukocyte differential count, glucose levels and blood creatining of hyperglycemic and ovalbumin sensitized *Mus musculus* Balb/c. Nusuntara Biosci..

[25] Harini M., Astirin O.P. (2009). Blood cholesterol levels of hypercholesterolemic rat (*Rattus norvegicus*) after VCO treatment. Nusant. Biosci..

[26] Dayrit C.S. (2003). Coconut oil: Atherogenic or not?. Philipp. J. Cardiol..

[27] Zakaria Z.A., Rofiee M.S., Somchit M.N., Zuraini A., Sulaiman M.R., Teh L.K., Salleh M.Z., Long K. (2011). Hepatoprotective activity of dried- and fermented-processed virgin coconut oil. Evid.-Based Complementary Altern. Med..

[28] Otuechere C.A., Madarikan G., Simisola T., Bankole O., Osho A. (2014). Virgin coconut oil protects against liver damage in albino rats challenged with the anti-folate combination, trimethoprim-sulfamethoxazole. J. Basic Clin. Physiol. Pharmacol..

[29] Babu A.S., Veluswamy S.K., Arena R., Guazzi M., Lavie C.J. (2014). Virgin coconut oil and its potential cardioprotective effects. Postgrad. Med..

[30] Gandotra S., Kour J., Van Der Waag A. (2014). Efficacy of adjunctive extra virgin coconut oil use in moderate to severe alzheimer’s disease. Int. J. Sch. Cogn. Psychol..

[31] Dayrit C.S. Coconut oil in health and disease: Its and monolaurins’s potential as cure for HIV/AIDS. Proceedings of the 36th Cocotech Meeting.

[32] Srivastava Y., Semwal A.D., Majumdar A. (2016). Quantitative and qualitative analysis of bioactive components present in virgin coconut oil. Cogent Food Agric..

[33] Bederson J.B., Pitts L.H., Tsuji M., Nishimura M.C., Davis R.L., Bartkowski H. (1986). Rat middle cerebral artery occlusion: Evaluation of the model and development of a neurologic examination. Stroke.

[34] Bailey S.A., Zidell R.H., Perry R.W. (2004). Relationships between organ weight and body/brain weight in the rat: What is the best analytical endpoint?. Toxicol. Pathol..

[35] Ben Othman M., Han J., El Omri A., Ksouri R., Neffati M., Isoda H. (2013). Antistress effects of the ethanolic extract from *Cymbopogon schoenanthus* growing wild in Tunisia. Evid.-Based Complementary Altern. Med..

[36] Narayanankutty A., Mukesh R.K., Ayoob S.K., Ramavarma S.K., Suseela I.M., Manalil J.J., Kuzhivelil B.T., Raghavamenon A.C. (2016). Virgin coconut oil maintains redox status and improves glycemic conditions in high fructose fed rats. J. Food Sci. Technol..

[37] Illam S.P., Narayanankutty A., Raghavamenon A.C. (2017). Polyphenols of virgin coconut oil prevent pro-oxidant mediated cell death. Toxicol. Mech. Methods.

[38] White A.R., Collins S.J., Maher F., Jobling M.F., Stewart L.R., Thyer J.M., Beyreuther K., Masters C.L., Cappai R. (1999). Prion protein-deficient neurons reveal lower glutathione reductase activity and increased susceptibility to hydrogen peroxide toxicity. Am. J. Pathol..

[39] Chaiswing L., Oberley T.D., Zhong W. (2014). Increasing discordant antioxidant protein levels and enzymatic activities contribute to increasing redox imbalance observed during human prostate cancer progression. Free. Radic. Biol. Med..

[40] Dickhout J.G., Lee R.M. (1998). Blood pressure and heart rate development in young spontaneously hypertensive rats. Am. J. Physiol..

[41] Nurul-Iman B.S., Kamisah Y., Jaarin K., Qodriyah H.M. (2013). Virgin coconut oil prevents blood pressure elevation and improves endothelial functions in rats fed with repeatedly heated palm oil. Evid. Based Complement Altern. Med..

[42] Júnior F.A.O., Ruiz C.R., de Oliveira Y., Barros M.A.V., Silva A.S., Santos M.S.B., Martins V.J.B., Balarini C.M., Braga V.A. (2021). Coconut Oil Supplementation Does Not Affect Blood Pressure Variability and Oxidative Stress: A Placebo-Controlled Clinical Study in Stage-1 Hypertensive Patients. Nutrients.

[43] Dyall S.C. (2015). Long-chain omega-3 fatty acids and the brain: A review of the independent and shared effects of EPA, DPA and DHA. Front. Aging Neurosci..

[44] Vitor R.J.S. (2018). Neuroprotective Effects of Virgin Coconut Oil in a Sprague Dawley Rat Ischemic Stroke Model. Master’s Thesis.

[45] Yamagata K., Tagami M., Watson R.R., Preedy V.R. (2015). Prevention of ischemia-induced neuronal apoptosis by vitamin E in stroke-prone spontaneously hypertensive rats: Cellular mechanisms of antioxidants. Bioactive Nutraceuticals and Dietary Supplements in Neurological and Brain Disease.

[46] Maalouf M., Rho J.M., Mattson M.P. (2009). The neuroprotective properties of calorie restriction, the ketogenic diet, and ketone bodies. Brain Res. Rev..

[47] Rui L. (2014). Energy metabolism in the liver. Compr. Physiol..

[48] Thaler S., Choragiewicz T.J., Rejdak R., Fiedorowicz M., Turski W.A., Tulidowicz-Bielak M., Zrenner E., Schuettauf F., Zarnowski T. (2010). Neuroprotection by acetoacetate and β-hydroxybutyrate against NMDA-induced RGC damage in rat—possible involvement of kynurenic acid. Graefe’s Arch. Clin. Exp. Ophthalmol..

[49] Mary S., Boder P., Rossitto G., Graham L., Scott K., Flynn A., Kipgen D., Graham D., Delles C. (2021). Salt loading decreases urinary excretion and increases intracellular accumulation of uromodulin in stroke-prone spontaneously hypertensive rats. Clin. Sci..

[50] Carnevale D., Lembo G. (2018). Heart, Spleen, Brain. Circulation.

[51] McIntosh T.K., Saatman K.E., Raghupathi R., Graham D.I., Smith D.H., Lee V.M., Trojanowski J.Q. (1998). The Dorothy Russell Memorial Lecture. The molecular and cellular sequelae of experimental traumatic brain injury: Pathogenetic mechanisms. Neuropathol. Appl. Neurobiol..

[52] Xing C., Arai K., Lo E.H., Hommel M. (2012). Pathophysiologic cascades in ischemic stroke. Int. J. Stroke.

[53] Hua F., Ma J., Ha T., Xia Y., Kelley J., Williams D.L., Kao R.L., Browder I.W., Schweitzer J.B., Kalbfleisch J.H. (2007). Activation of Toll-like receptor 4 signaling contributes to hippocampal neuronal death following global cerebral ischemia/reperfusion. J. Neuroimmunol..

[54] Ning R., Chopp M., Zacharek A., Yan T., Zhang C., Roberts C., Lu M., Chen J. (2014). Neamine induces neuroprotection after acute ischemic stroke in type one diabetic rats. Neuroscience.

[55] Broughton B.R.S., Reutens D.C., Sobey C.G. (2009). Apoptotic mechanisms after cerebral ischemia. Stroke.

[56] Diestro J.D.B., Omar A.T., Climacosa F.M.M., Mondia M.W.L., Arbis C.C.H., Collantes T.M.A., Khu K.J.O., Roxas A.A., Estacio M.A.C. (2021). Virgin coconut oil attenuates deficits in rats undergoing transient cerebral ischemia. Acta Med. Philipp..

[57] Li K., Li J., Zheng J., Qin S. (2019). Reactive Astrocytes in Neurodegenerative Diseases. Aging Dis..

